# Discovery and identification of a novel yeast species, Hanseniaspora drosophilae sp. nov., from Drosophila in Okinawa, Japan

**DOI:** 10.1099/ijsem.0.006661

**Published:** 2025-02-03

**Authors:** Taisuke Seike, Hiroki Takekata, Keiko Kono, Natsue Sakata, Hazuki Kotani, Chikara Furusawa, Fumio Matsuda

**Affiliations:** 1 Department of Bioinformatics Engineering, Graduate School of Information Science and Technology Osaka University, 1-5 Yamadaoka, Suita, Osaka, 565-0871, Japan; 2 Center for Biosystems Dynamics Research, RIKEN, 6-2-3 Furuedai, Suita, Osaka, 565-0874, Japan; 3 Organization for Research Promotion, University of the Ryukyus, 1 Senbaru, Nishihara, Okinawa, 903-0213, Japan; 4 Okinawa Institute of Science and Technology Graduate University, 1919-1 Tancha, Onna, Okinawa 904-0495, Japan; 5 Universal Biology Institute, The University of Tokyo, 7-3-1 Hongo, Tokyo, 113-0033, Bunkyo-ku, Japan

**Keywords:** *Drosophila*, *Hanseniaspora drosophilae *sp. nov., Japan, Okinawa, yeasts

## Abstract

*Hanseniaspora*, a genus of yeasts in which many species reproduce sexually, has attracted the attention of researchers because of its prevalence in diverse ecological niches. Building on our extensive collection efforts since 2020, three previously unknown yeast strains from wild *Drosophila* species trapped in ripe bananas in Okinawa, Japan, were isolated. Using a multifaceted approach, including physiological assessments and sequence analysis of the D1/D2 domain of the 26S LSU rRNA gene and the internal transcribed spacer (ITS) region, it was revealed that these strains are novel members of the genus *Hanseniaspora*. The three strains, JCM 36741^T^, JCM 36742 and JCM 36748, had identical sequences in their respective D1/D2 and ITS regions, justifying their classification as a single species. Moreover, the new species exhibited a remarkable degree of sequence divergence from its closest relatives, differing by 7 nucleotide substitutions (1.28%) in the D1/D2 domain, 29 nucleotide substitutions and 4 gaps (4.08%) in the ITS regions. These substantial sequence differences highlight the distinctiveness of this novel species in the genus *Hanseniaspora*. Further analysis revealed physiological characteristics that distinguished the new species from its closest relative, *Hanseniaspora hatyaiensis* (nom. inval.). These findings culminated in the proposed name *Hanseniaspora drosophilae* sp. nov., which recognizes the unique ecological niche within the *Drosophila* microbiota. By uncovering this novel species, this study not only adds to the growing body of knowledge on yeast diversity but also sheds light on the intricate ecological relationships that shape microbial communities. The implications of this discovery extend beyond taxonomic boundaries, inviting further exploration of the evolutionary dynamics and ecological significance of yeast–fly interactions. We propose accommodating *H. drosophilae* sp. nov. in the genus *Hanseniaspora* with JCM 36741^T^ as the holotype. The MycoBank accession number is MB 853822.

## Introduction

Zikes proposed the genus *Hanseniaspora* to encompass sexually reproducing yeasts, with *Hanseniaspora valbyensis* designated as the type species, while its anamorphic counterpart is known as *Kloeckera* [1]. However, *Hanseniaspora* and *Kloeckera* share notable morphological and biochemical characteristics [1,3]. With recent taxonomic changes, *Kloeckera* species have been reclassified to the genus *Hanseniaspora*. This change was in accordance with the new International Code of Nomenclature for algae, fungi and plants as it ranks the teleomorph stage over the anamorph stage [4]. In *The Yeasts*, fifth edition, the genus *Hanseniaspora* includes 11 species [1]. However, subsequent studies have expanded this roster and incorporated species such as *Hanseniaspora thailandica* [5], *Hanseniaspora singularis* [5], *Kloeckera hatyaiensis* (*Hanseniaspora hatyaiensis*) [5], *Kloeckera taiwanica* (*Hanseniaspora taiwanica*) [6], *Hanseniaspora nectarophila* [7], *Hanseniaspora jakobsenii* [8], *Hanseniaspora gamundiae* [4], *Hanseniaspora mollemarum* [9], *Hanseniaspora terricola* [10], *Hanseniaspora smithiae* [11] and *Hanseniaspora menglaensis* [12], effectively doubling the number of known species within the genus.

*Hanseniaspora* species are a group of yeast species present in various ecological niches ranging from flowers and fruits to rotting wood and soil and thrive on insect-associated fruit and flowers [4,8,10]. Notably, *Hanseniaspora* (*Kloeckera*) are typically used in the fermentation process of grapes, adding a rich palette of colours and flavours to wine [14]. *Drosophila* is a genus of fruit flies that are attracted to specific fermented substrates present on fruits and sap fluxes of trees [15]. These flies interact with the microbiota in these environments [15,16], facilitating the transportation of *Hanseniaspora* species to new habitats. This intricate relationship highlights the importance of further characterization of *Hanseniaspora* species for both ecological understanding and industrial applications, such as winemaking [17].

Therefore, this study explores the physiological characteristics and phylogenetic relationships of *Hanseniaspora* species isolated from Okinawa Prefecture, Japan, and their interactions with *Drosophila*. Furthermore, this research introduces a novel species, *Hanseniaspora drosophilae* sp. nov., thereby adding a new dimension to the *Hanseniaspora* clade and shedding light on its intricate ecological interactions.

## Methods

### Yeast isolation and identification

*Drosophila* species used in the present study were collected from 2020 to 2022 using traps at the Nishihara Campus of the University of the Ryukyus (26° 14′ N 127° 46′ E) in Nishihara, Okinawa Prefecture, Japan, located on the main island of Okinawa in the Ryukyu Archipelago, and at the Okinawa Institute of Science and Technology Graduate University (26° 46′ N 127° 83′ E) in Okinawa Prefecture, Japan. Hundreds of flies were collected from the Nishihara Campus, and ~40 flies were collected from the Okinawa Institute of Science and Technology Graduate University.

The trap consisted of a milk carton suspended by a rope from tree branches ~2 m above the ground, with a piece of peeled ripe banana placed inside. The milk carton was covered with an aseptic plastic bag to trap visiting *Drosophila*, and the traps were retrieved after 2–3 days. Although the possibility of yeast originating from the bait (banana) cannot be completely ruled out, the flies were rinsed with sterilized water before homogenization to minimize external contamination. This method did not ensure the complete removal of yeast cells adhering to the flies’ exteriors, but it was assumed that most isolated yeasts were associated with the flies either externally or internally. The potential transfer of yeasts from the bait to the flies during trapping and subsequent washing was noted as a limitation of this study.

The flies were placed in sterile 1.5 ml tubes in groups of approximately ten flies and thoroughly rinsed with sterilized water to clean their body surfaces. Flies, including their guts and crops, were homogenized using a homogenizer pestle (INA OPTIKA, Osaka, Japan). The resulting samples were diluted with sterilized water and plated on yeast extract agar (YEA) plates (0.5% yeast extract, 3% peptone and 1.5% agar) or yeast extract–peptone dextrose (YPD) plates (1% yeast extract, 2% peptone, 2% d-glucose and 2% agar). To inhibit bacterial growth, 100 µg ml^−1^ ampicillin and 100 µg ml^−1^ chloramphenicol were added to the plates, which were incubated for 2–3 days at 30 °C.

The acquired yeast colonies were examined under an upright microscope (CX23, Olympus, Tokyo, Japan) and categorized based on colour, surface appearance and morphology. All purified yeast strains were suspended in 15% glycerol stock and maintained at −80 °C until required for use.

### DNA sequencing and phylogenetic analysis

Genomic DNA was extracted from cultures grown overnight in YPD medium (1% yeast extract, 2% peptone and 2% d-glucose) using the Wizard Genomic DNA Purification Kit (Promega, Madison, WI, USA). The sequences of the D1/D2 domain of the 26S LSU rRNA gene and the internal transcribed spacer (ITS) region were determined by PCR amplification of genomic DNA extracted from yeast cells. The PCR amplification of the D1/D2 domain of the 26S LSU rDNA was performed using KOD FX Neo DNA polymerase (TOYOBO, Tokyo, Japan) with the forward primer NL1 (5′-GCATATCAATAAGCGGAGGAAAAG-3′) and the reverse primer NL4 (5′-GGTCCGTGTTTCAAGACGG-3′) [18]. The ITS regions were amplified using the forward primer ITS1 (5′-TCCGTAGGTGAACCTGCGG-3′) and the reverse primer ITS4 (5′-TCCTCCGCTTATTGATATGC-3′) [19]. Sanger sequencing (Eurofins Genomics sequencing service, NY, USA) was used to determine the nucleotide sequence of each PCR product using the primers NL1 and NL4 for the D1/D2 domain and ITS1 and ITS4 for the ITS region.

**Fig. 1.**
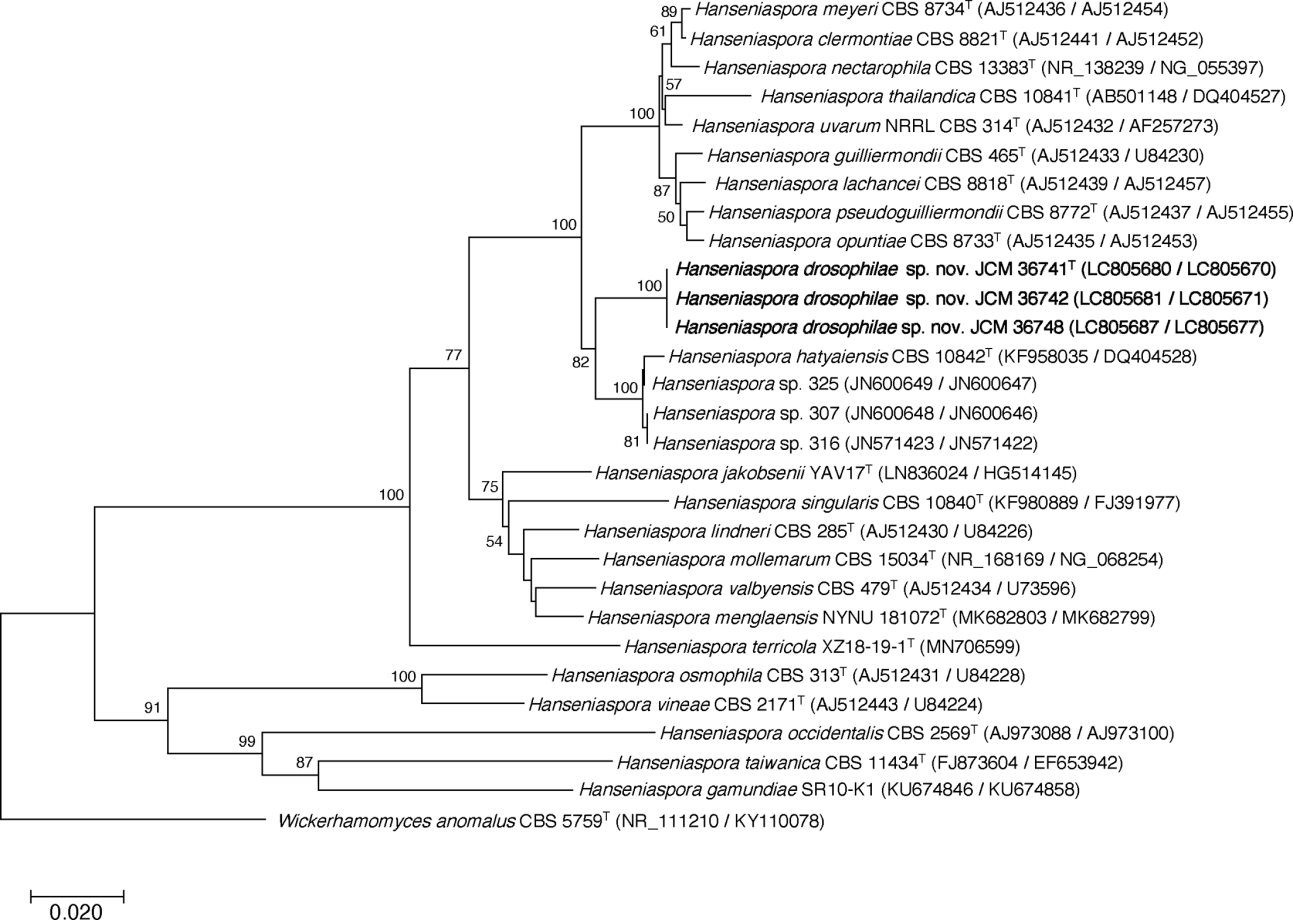
Phylogenetic tree of *Hanseniaspora drosophilae* sp. nov. and related species based on NJ analysis of the concatenated sequences of the ITS and D1/D2 domain of the LSU rRNA gene. The tree was constructed using the NJ method in the mega X software (v10.1.8). The numbers on the branches represent the frequencies with which a given branch appeared in the 10 000 bootstrap replicates. Bootstrap values >50% are shown. *Wickerhamomyces anomalus* CBS 5759^T^ (NR_111210/KY110078) was selected as the outgroup. Scale bar, 0.020 substitutions per site.

The resulting sequences were aligned using the multiple sequence comparison by log-expectation algorithm [20] and assembled using the mega X software (v10.1.8) [21] to obtain complete gene sequences. Phylogenetic analysis based on the concatenated sequences of the ITS and D1/D2 domains of the LSU rRNA gene was performed in mega X using the neighbour-joining (NJ) method with Kimura’s two-parameter distance correction (Fig. 1). Confidence levels of the clades were derived from 10 000 bootstrap replicates [22]. *Wickerhamomyces anomalus* CBS 5759^T^ (KY110078/NR_111210) was used as the outgroup for this analysis.

Draft genome sequences were generated using the Illumina MiSeq system (Illumina, San Diego, CA, USA). A 300 bp paired-end library was constructed according to the Illumina protocol and sequenced using Illumina MiSeq, resulting in at least 200-fold coverage. Sequence reads were subjected to *de novo* assembly using SPAdes Genome Assembler (v3.15.5), an open-source *de novo* sequencing tool (https://bio.tools/spades). Illumina MiSeq genome data were used to validate the D1/D2 and ITS sequences.

The draft genome sequence of *H. drosophilae* sp. nov. (JCM 36741^T^) was deposited in the NCBI database under accession numbers BAAGDS010000001–BAAGDS010002311. Using the assembled genome data, the G+C content and genome size were calculated, with the values of ~26.7% and 9.6 Mb, respectively. Comparative analysis was conducted using average nucleotide identity (ANI) to measure the similarity between *H. drosophilae* sp. nov. and 17 other representative *Hanseniaspora* species [23]. Genomic sequences of these species were retrieved from the NCBI for Biotechnology Information database. ANI calculations were performed using OrthoANI [24], and the results are shown in Fig. S1, available in the online Supplementary Material. The G+C content and genome size of the representative genomes are summarized in Table S1, enabling a direct comparison with * H. drosophilae* sp. nov. These analyses supported the taxonomic distinction between the new species.

### Physiological and morphological characterization

Physiological and morphological characterizations were performed on the novel yeast strains, according to standard procedures described by Kurtzman *et al*. [25], and supplemented by recent studies on *Hanseniaspora* species [10,12]. The investigation included the observation of pseudo-hyphae, true hyphae and ascospores incubated at 25 °C for up to 4 weeks and cultivated on various media types, including 5% malt extract agar (MEA; 5% malt extract, 0.5% peptone and 1.5% agar), corn meal agar (CMA), potato dextrose agar (PDA), yeast morphology agar (YMoA), yeast carbon base ammonium sulphate agar (1.1% yeast carbon base, 0.01% ammonium sulphate and 1.8% agar), V8 juice agar (V8A; 20% V8 juice, 0.2% calcium carbonate and 1.5% agar) and diluted V8A (1 : 9). Assimilation tests for carbon and nitrogen sources were performed on agar plates within 7 days at 25 °C. Carbohydrate fermentation was assessed in a 5 ml liquid medium using Durham fermentation tubes at 25 °C for up to 7 days. Cycloheximide resistance was determined using YPD plates. Diazonium blue B (DBB) reactions were assessed using solid media. In addition, growth at different temperatures (8, 10, 12, 15, 19, 25, 30, 35, 37 and 40 °C) was evaluated within 7 days of cultivation on YPD plates. Yeast cells from the agar plates were directly observed without washing or fixation. Imaging was performed using cellSens standard imaging software (Olympus), with a differential interference contrast microscope (BX53F2; Olympus) equipped with an Olympus UPlanSApo 60×/1.35na oil objective and camera (ORCA-spark, Hamamatsu Photonics, Hamamatsu, Japan).

## Results and discussion

### Novel species identification and delineation

Wild *Drosophila* species are attracted to fermented substrates, such as ripe grapes, where they feed and lay eggs [16,26]. *Hanseniaspora* species can grow rapidly on rotting fruit, allowing them to dominate the surface microbiota of the fruit [27].

**Fig. 2.**
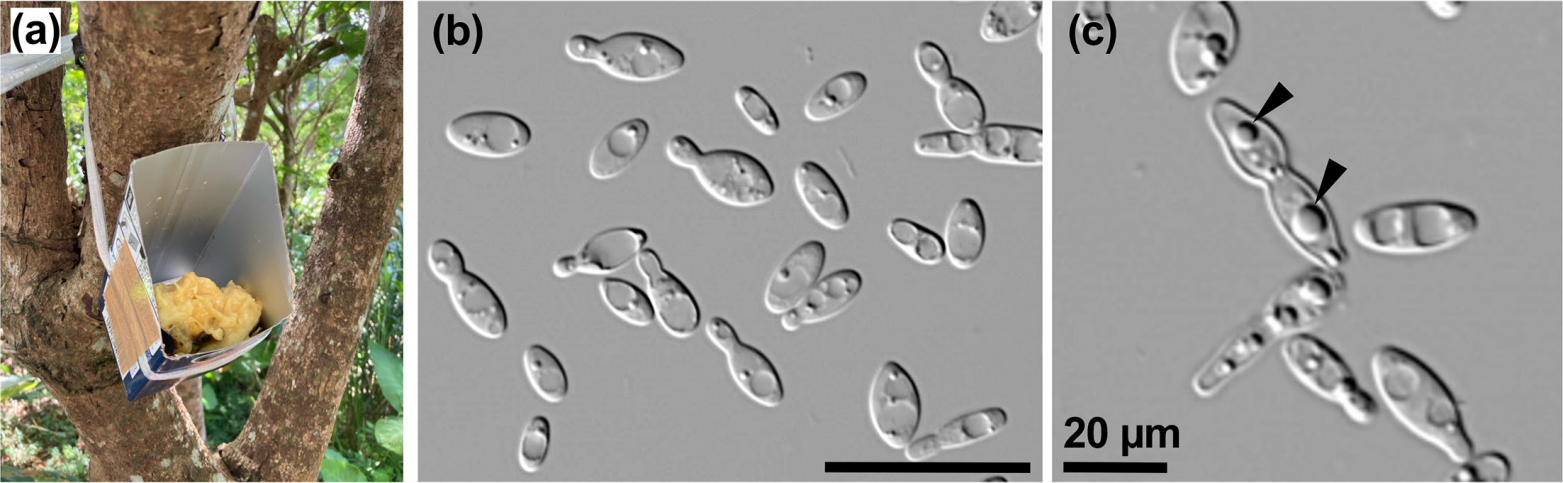
Microscopic observation of the novel yeast species. (**a**) Banana trap to collect *Drosophila* species and (**b**) budding cells of *H. drosophilae* sp. nov. JCM 36741^T^ on YM agar after 3 days at 25 °C. Cells are lemon-shaped. (**c**) Ascospores (arrow) of JCM 36741^T^ on YMoA after 1 week. Scale bar, 20 µm.

In this study, wild yeast species from hundreds of wild *Drosophila* trapped using ripe bananas (Fig. 2a) in Okinawa between 2020 and 2022 were isolated. Although the use of bananas as bait introduces the possibility of yeast originating from the fruit itself or the surrounding environment, rinsing and homogenization methods have been designed to minimize external contamination and focus on yeasts associated with *Drosophila*. Thirty-three yeast strains were obtained, among which the following *Hanseniaspora* species were isolated and identified: *H. drosophilae* sp. nov. [TN337 (JCM 36741^T^), TN350 (JCM 36742) and TN1065 (JCM 36748)], *Hanseniaspora pseudoguilliermondii*, *H. valbyensis* and *Hanseniaspora uvarum* (Table 1). Notably, *H. drosophilae* sp. nov. was isolated twice over 2 years from different areas of Okinawa Prefecture. These observations are consistent with our interpretation that these species are likely distributed by *Drosophila* in the Okinawa area, although the possibility of other origins such as bait cannot be completely excluded.

**Table 1.** List of yeast species isolated from *Drosophila* in this study

Date of collection	Sampling area	Isolated yeast species
September 2020	The Nishihara Campus of the University of the Ryukyus(26° 14′ N 127° 46′ E)	*Candida orthopsilosis*
		*Candida tropicalis*
		*Hanseniaspora drosophilae* sp. nov. (TN337, TN350)
		*Hanseniaspora valbyensis*
		*Kurtzmaniella natalensis*
		*Kurtzmaniella quercitrusa*
		*Pichia fermentans*
		*Pichia kudriavzevii*
		*Starmerella bacillaris*
		*Sungouiella intermedia*
		*Trichosporon coremiiforme*
		*Wickerhamomyces onychis*
		*Wickerhamomyces pijperi*
July 2022	The Nishihara Campus of the University of the Ryukyus (26° 14′ N 127° 46′ E)	*Candida conglobata*
		*Candida sorboxylosa*
		*Clavispora fructus*
		*Debaryomyces hansenii*
		*Debaryomyces nepalensis*
		*Hanseniaspora uvarum*
		*Hyphopichia burtonii*
		*Kodamaea ohmeri*
		*Kurtzmaniella natalensis*
		*Lachancea fermentati*
		*Martiniozyma asiatica*
		*Meyerozyma neustonensis*
		*Pichia kluyveri*
		*Pichia kudriavzevii*
		*Saturnispora diversa*
		*Sungouiella intermedia*
		*Torulaspora delbrueckii*
		*Wickerhamiella azyma*
September 2022	The Okinawa Institute of Science and Technology Graduate University (26° 46′ N 127° 83′ E)	*Candida sorboxylosa*
		*Hanseniaspora drosophilae* sp. nov. (TN1065)
		*Hanseniaspora pseudoguilliermondii*
		*Pichia fermentans*
		*Pichia kudriavzevii*
		*Trichosporon asahii*
		*Trichosporon insectorum*

These lists include the date of collection, sampling area and isolated yeast species. Species identification of the yeast-like colonies grown on either YEA or YPD plates, on which the *Drosophila* extract was plated, was performed by determining the sequence of the D1/D2 region.

Shared sequences between the acquired three strains (JCM 36741^T^, JCM 36742 and JCM 36748) were identified in their D1/D2 and ITS regions. Because these strains are thought to belong to the same species, JCM 36741^T^ was selected as a candidate for a novel yeast species and therefore further investigated. A standard nucleotide blast in the GenBank database revealed that JCM 36741^T^ shared 98.72% sequence identity with *Hanseniaspora* sp. 325 (JN600647), with seven nucleotide substitutions (565/572), and no gaps in the D1/D2 domain. The second closest species is *K. hatyaiensis* (*H. hatyaiensis*) FRN2-2 (LC387262), which shared eight nucleotide differences (564/572) with JCM 36741^T^. With respect to the ITS regions, sequence analysis showed that JCM 36741^T^ differed from *Hanseniaspora* sp. 307 (JN600648) and *Hanseniaspora* sp. 316 (JN571423) with 95.92% identity (682/711) and four gaps, *H. hatyaiensis* CBS 10842 with 95.75% identity (676/706) and four gaps and *Hanseniaspora* sp. 325 (JN600649) with 95.64% identity (680/711) and four gaps (query coverage of 99–100%). In the genus *Hanseniaspora*, the mutation rates for sequence substitutions in the D1/D2 and ITS regions were lower than those in other genera [28]. Considering these results and previous examples of distinct *Hanseniaspora* species, our results indicated a substantial degree of sequence divergence, sufficient to differentiate *Hanseniaspora* species at the species level.

The phylogenetic tree was constructed based on concatenated sequences of the ITS and D1/D2 domains of the LSU rRNA gene by using the NJ methods with 10 000 random bootstrap replicates. Phylogenetic analysis revealed that the three strains (JCM 36741^T^, JCM 36742 and JCM 36748) belonged to the genus *Hanseniaspora* and formed a considerably supported subclade (bootstrap value of 80%), including *H. hatyaiensis* and some registered *Hanseniaspora* spp. (Fig. 1).

In addition to phylogenetic analyses based on the D1/D2 and ITS regions, the genome of JCM 36741^T^ was sequenced using the Illumina MiSeq platform. Preliminary genome analysis revealed a G+C content of ~26.7% and a genome size of ~9.6 Mb. Notably, these values were slightly different from those of *H. hatyaiensis*, which has a G+C content of ~34.9% [5] and a genome size of 8.9 Mb (Table S1). Based on existing studies, *Hanseniaspora* species typically exhibit G+C contents ranging from 26.0 to 37.0% and genome sizes from 8.7 to 11.5 Mb. Thus, JCM 36741^T^ has a relatively lower G+C content and genome size comparable to the average of other *Hanseniaspora* species. These differences in genome size and G+C content may reflect underlying genomic and ecological adaptations, although further detailed analyses are necessary to draw specific conclusions regarding the implications of these differences.

Comparative genomic analysis was conducted to further investigate genetic relationships. ANI values are expected to become a new standard for defining yeast species based on their genetic divergence. To validate the genetic relationships, sequence reads were obtained using MiSeq and assembled to generate a draft genome sequence of JCM 36741^T^. Comparative analysis of whole-genome sequences from representative *Hanseniaspora* species confirmed that JCM 36741^T^ was the species most closely related genetically to *H. hatyaiensis* (Fig. S1). This result is consistent with the phylogenetic relationship based on the ITS–D1/D2 sequences (Fig. 1). Taken together, the genetic analysis showed that the isolated species belonged to the *Hanseniaspora* clade and that the most closely related species among the accepted species was likely *H. hatyaiensis*.

Although *H. hatyaiensis* is listed as a recognized species in GenBank and has been formally described in the literature [5], its nomenclature is considered invalid (nom. inval.). This may result from procedural issues such as the preservation of the type strain that does not meet the requirements of the International Code of Nomenclature for algae, fungi, and plants. However, * H. hatyaiensis* (nom. inval.) remains a significant taxonomic reference because it is one of the species most closely related to JCM 36741^T^.

Next, the physiological characteristics (fermentation ability of certain carbons, assimilation of different carbons/nitrogen and growth tests under different culture conditions) of the isolated species were investigated using all three strains (JCM 36741^T^, JCM 36742 and JCM 36748). In the present study, these three novel strains were distinguished from *H. hatyaiensis* (nom. inval.) based on their phenotypic characteristics (Table 2). Compared to the type strain ST-476^T^ of *H. hatyaiensis*, JCM 36741^T^, JCM 36742 and JCM 36748 assimilated inulin (weak), d-sucrose, d-raffinose (slow) and d-cellobiose as carbon sources, whereas *H. hatyaiensis* showed almost no assimilations [5]. Conversely, JCM 36741^T^, JCM 36742 and JCM 36748 did not assimilate 2-keto-d-gluconate, unlike *H. hatyaiensis*. JCM 36741^T^, JCM 36742 and JCM 36748 assimilated ethylamine but did not assimilate nitrite and l-lysine as nitrogen sources. In addition, JCM 36741^T^, JCM 36742 and JCM 36748 cells were grown on YPD plates containing 0.1% cycloheximide, which typically inhibits the growth of most yeast cells. This phenotype is similar to that observed in *H. uvarum* [1]. The growth of JCM 36748 occurred at 35 °C, similar to that of *H. guilliermondii*, *Hanseniaspora opuntia*e and *H. pseudoguilliermondii* [1], whereas the other two strains, JCM 36741^T^ and JCM 36742, did not grow at this temperature. Moreover, JCM 36741^T^, JCM 36742 and JCM 36748 demonstrated normal growth at 10 °C, and JCM 36748 survived even at 8 °C for 7 days. This species exhibited vitamin auxotrophy and may require pantothenate, niacin, pyridoxine and thiamine similar to *H. hatyaiensis* [5]. Based on these physiological characteristics, the novel species were distinct from that of its closest species. Therefore, * H. drosophilae* sp. nov. was presented as the proposed name for this yeast strain.

**Table 2.** Comparison of physiological characteristics between *Hanseniaspora drosophilae* sp. nov. and the related species *Hanseniaspora hatyaiensis* (nom. inval.)

Physiological characteristics	*H. drosophilae* sp. nov.			*H. hatyaiensis*
Strains	JCM 36741^T^	JCM 36742	JCM 36748	ST-476^T^
Assimilation of carbon compounds				
Inulin	w	w	w	−
Sucrose	+	+	+	−
Raffinose	s/+	s/+	s/+	−
Cellobiose	+	+	+	−
2-keto-d-gluconate	−	−	−	+
Assimilation of nitrogen compounds				
Nitrite	−	−	−	+
l-Lysine	−	−	−	+
Ethylamine	+	+	+	−
Others				
Amino acid-free	+	+	+	nd
Cycloheximide 0.01%	+	+	+	−
Cycloheximide 0.1%	+	+	+	−
Growth at 8 °C	−	−	s/+	nd
Growth at 35 °C	−	−	+	−

*Hanseniaspora drosophilae* sp. nov.: data obtained from JCM 36741T, JCM 36742 and JCM 36748; *H. hatyaiensis*: data for type strain ST-476T [5].

Growth reactions: +, positive; −, negative; w, weak growth; s, slow growth; nd, no data.

During the study period, *Hanseniaspora* species were prevalent among yeasts harboured by *Drosophila*, with *H. opuntiae* and * H. uvarum* being particularly common. Interestingly, *H. drosophilae* sp. nov. has thus far been exclusively associated with *Drosophila* in Okinawa, with no instances of its isolation from other sources in the region, including various plants such as *Bidens pilosa* var. *radiata* Sch. Bip [29], and other places despite our extensive sampling efforts. Although our data provide compelling evidence of specific yeast-*Drosophila* interactions, the generalizability of these findings remains uncertain. It seems plausible that the observed association between *Drosophila* and *H. drosophilae* sp. nov. is coincidental and lacks broader ecological implications.

Another yeast species, *Pichia kluyveri*, was isolated. Interestingly, this species tends to be highly attractive to *Drosophila melanogaster* [30], which may contribute to its frequent associations, with *Drosophila*. Therefore, the findings of this study suggest that wild yeast species such as *Hanseniaspora* and *Pichia* are often associated with fruiting plants and have been frequently isolated from wild *Drosophila*. However, the extent and ecological significance of these associations require further investigation because the observed interactions may not represent generalizable patterns across all *Drosophila* populations or habitats.

Our previous study demonstrated that many fission yeast strains of *Schizosaccharomyces japonicus* isolated from *Drosophila* exhibited tolerance to high-temperature stress [31]. Additionally, experimental evidence has suggested that the spores of the budding yeast, *Saccharomyces cerevisiae*, which is known for its resistance to high-temperature stress, can survive passage through the gut of *D. melanogaster* [32]. These findings imply that heat-tolerant species or spores may have a competitive advantage within *Drosophila* microbiota. However, it is important to note that among the strains of *H. drosophilae* sp. nov., only JCM 36748 demonstrated high-temperature tolerance, while JCM 36741^T^ and JCM 36742 did not grow at 35 °C. This variation suggests that within *H. drosophilae* sp. nov., strain-specific differences may exist in their interactions with *Drosophila* and adaptation to high-temperature environments. To gain a deeper understanding of the nature of the interactions between *Drosophila* and yeasts, it is imperative to conduct rigorous experiments that systematically test hypotheses regarding the fitness benefits and costs associated with specific associations.

### Description of *Hanseniaspora drosophilae* sp. nov. Seike, Takekata and Kono

*Hanseniaspora drosophilae* (dro.so'phi.lae. N.L. gen. n. *drosophilae*, of the fruit fly genus *Drosophila*).

After 3 days of growth at 25 °C on YM agar, the cells are primarily apiculate, a characteristic feature of the genus, with some younger cells appearing ovoid (2.6–5.2×4.8–13.2 µm; Fig. 2b). Bipolar budding is observed. On the YM medium, the streak cultures are white, smooth, glossy and flat to slightly raised in the centre, with entire margins. No pseudo-hyphae are observed after growth on any media. Ascospores (1.5–2.0 µm) were formed on the CMA, MEA, PDA and YMoA (Fig. 2c) at 25 °C for 1 week. The asci, which measured 11.1–17.2 µm, were observed under these conditions. However, no asci are observed after growth on other media.

Fermentation is positive for d-glucose but negative for d-galactose, d-sucrose, d-maltose, d-lactose, d-raffinose and d-trehalose. Carbon assimilation is positive for d-glucose, inulin (weak), d-sucrose, d-raffinose, d-cellobiose, d-salicin, d-gluconate and d-glucono-1,5-lactone, and negative for d-melibiose, d-galactose, lactose, d-trehalose, d-maltose, d-melezitose, methyl-α-d-glucoside, soluble starch, l-sorbose, l-rhamnose, d-xylose, l-arabinose, d-arabinose, d-ribose, methanol, ethanol, glycerol, erythritol, ribitol, galactitol, d-mannitol, d-glucitol, *myo*-inositol, dl-lactate, succinate, citrate, d-glucosamine, *N*-acetyl-d-glucosamine, hexadecane, 2-keto-d-gluconate, xylitol and d-glucuronate. Nitrogen assimilation is positive for cadaverine and ethylamine but negative for nitrate, nitrite and l-lysine. Growth in the amino acid-free medium is positive, whereas that in the vitamin-free medium is negative. Additionally, it is negative in the presence of 50% (w/v) glucose and 10% (w/v) sodium chloride plus 5% (w/v) glucose. Growth is observed even in the presence of 0.1% cycloheximide, as well as at 8 (variable), 10, 12, 15, 19, 25, 30 and 35 °C (variable) but not at 37, 40 and 45 °C. The DBB test results are negative.

The strain JCM 36741^T^ is designated as the holotype of *H. drosophilae* sp. nov. It is isolated from the fruit fly *Drosophila* trapped by ripe bananas collected from the Nishihara campus of the University of the Ryukyus on Okinawa Main Island, Japan, and is preserved in a metabolically inactive state at the Japan Collection of Microorganisms (JCM). The ex-type culture is preserved in Westerdijk Institute, The Netherlands, as CBS 18611, and the National Institute of Technology and Evaluation, Japan, as NBRC 116652. The GenBank/EMBL/DDBJ accession numbers for the sequences of the D1/D2 domain of the 26S LSU rRNA gene and the ITS region determined in this study are LC805670 and LC805680, respectively. Strains JCM 36742 and JCM 36748 are also designated as *H. drosophilae* sp. nov. and are isolated from *Drosophila* fry from the Nishihara campus of the University of the Ryukyus on Okinawa Main Island and Okinawa Institute of Science and Technology Graduate University, Japan, respectively. They are preserved in metabolically inactive states, such as JCM 36742 (=CBS 18612=NBRC 116653) and JCM 36748 (=CBS 18613=NBRC 116659). The GenBank/EMBL/DDBJ accession numbers for the sequences of the D1/D2 domain of the 26S LSU rRNA gene and the ITS region determined in this study are LC805671 and LC805681 for JCM 36742 and LC805677 and LC805687 for JCM 36748. The MycoBank accession number is MB 853822.

## Supplementary material

10.1099/ijsem.0.006661 Uncited Supplementary Material 1.

## Abbreviations

ANI: average nucleotide identity
CMA: corn meal agar
DBB: diazonium blue B
ITS: internal transcribed spacer
JCM: Japan Collection of Microorganisms
MEA: malt extract agar
NJ: neighbour-joining
PDA: potato dextrose agar
V8A: V8 juice agar
YCBAS: yeast carbon base ammonium sulphate
YEA: yeast extract agar
YMoA: yeast morphology agar
YPD: yeast extract–peptone dextrose
