# Supplementary material for: Discovery and identification of a novel yeast species, Hanseniaspora drosophilae sp. nov., from Drosophila in Okinawa, Japan
Source: Int J Syst Evol Microbiol. 2025 Feb 3;75(2):006661. doi: 10.1099/ijsem.0.006661 (PMC12281848; doi:10.1099/ijsem.0.006661)
Supplement: Uncited Supplementary Material 1. [file ijsem-75-06661-s001.pdf]

Fig. S1

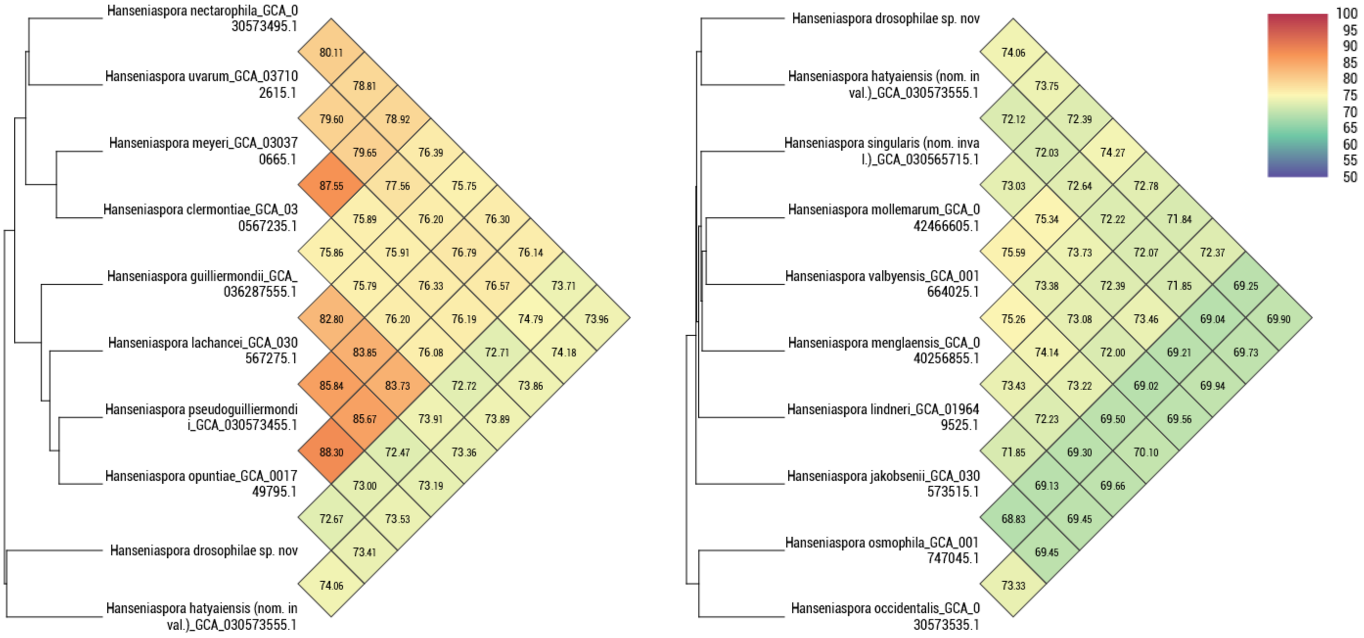

Heatmap generated with OrthoANI values calculated using the OAT software from representative *Hanseniaspora* species.

Hanseniaspora drosophilae sp. nov. (JCM 36741^{T}) and 17 other *Hanseniaspora* species are included in the nomenclature. The genome data used for the analysis were retrieved from the NCBI database, and a detailed list of GenBank assembly numbers and associated metadata is provided in Table S1.

Table S1

Genomic sequences of *Hanseniaspora* species retrieved from the NCBI for Biotechnology Information database.

	GenBank assembly	Genome size (Mb)	Number of chromosomes	Number of scaffolds	Number of contigs	GC precent	Genome coverage	Assembly level
Hanseniaspora meyeri	GCA_030370665.1	8.8	7	7	7	37.0	100.0x	Complete Genome
Hanseniaspora clermontiae	GCA_030567235.1	8.7		267	314	37.0	54.0x	Scaffold
Hanseniaspora nectarophila	GCA_030573495.1	8.7		389	393	34.0	237.3x	Scaffold
Hanseniaspora uvarum	GCA_037102615.1	9.1			13	32.0	298.3x	Contig
Hanseniaspora guilliermondii	GCA_036287555.1	9.1	8	8	8	31.0	46.0x	Complete Genome
Hanseniaspora lachancei	GCA_030567275.1	8.9		432	447	35.0	165.2x	Scaffold
Hanseniaspora pseudoguilliermondii	GCA_030573455.1	8.8		171	243	34.5	76.2x	Scaffold
Hanseniaspora opuntiae	GCA_001749795.1	8.8		17	66	35.0	60.0x	Scaffold
***Hanseniaspora hatyaiensis* (nom. inval.)**	GCA_030573555.1	9.6		259	304	36.5	114.8x	Scaffold
Hanseniaspora jakobsenii	GCA_030573515.1	11.0		464	618	30.0	70.8x	Scaffold
***Hanseniaspora singularis* (nom. inval.)**	GCA_030565715.1	8.9		294	437	26.0	80.1x	Scaffold
Hanseniaspora lindneri	GCA_019649525.1	10.8		282	326	36.0	87.6x	Scaffold
Hanseniaspora mollemarum	GCA_042466605.1	9.2	8	8	8	35.0	113.0x	Complete Genome
Hanseniaspora valbyensis	GCA_001664025.1	11.5		647	1345	26.5	53.0x	Scaffold
Hanseniaspora menglaensis	GCA_040256855.1	9.5	7	7	7	30.5	260.0x	Complete Genome
Hanseniaspora osmophila	GCA_001747045.1	11.5		17	899	37.0	60.0x	Scaffold
Hanseniaspora occidentalis	GCA_030573535.1	11.3		376	577	35.0	94.4x	Scaffold
***Hanseniaspora drosophilae* sp. nov.**		9.6			2311	26.7	264.5x	Contig
